# The Role of Immunotherapy and Radiation Therapy in the Treatment of Breast Cancer

**DOI:** 10.3390/biomedicines13092209

**Published:** 2025-09-09

**Authors:** Drishti Panse, Kristin Hsieh, Danielle Arons, Juliana Runnels, Monica Wassel, Anuja Shah, Rima Patel, Amy Tiersten, Anthony D. Nehlsen, Deborah Marshall, Robert M. Samstein, Sheryl Green, Julie Bloom

**Affiliations:** 1 Department of Radiation Oncology, Icahn School of Medicine at Mount Sinai, New York, NY 10029, USA; kristin.hsieh@mountsinai.org (K.H.); danielle.arons@mountsinai.org (D.A.); juliana.runnels@mountsinai.org (J.R.); monica.wassel@mountsinai.org (M.W.); anuja.shah@mountsinai.org (A.S.); anthony.nehlsen@mountsinai.org (A.D.N.); deborah.marshall@mountsinai.org (D.M.); sheryl.green@mountsinai.org (S.G.); julie.bloom@mountsinai.org (J.B.); 2 Department of Hematology/Oncology, Department of Medicine, Icahn School of Medicine at Mount Sinai, New York, NY 10029, USA; rima.patel2@mssm.edu (R.P.); amy.tiersten@mssm.edu (A.T.)

**Keywords:** immunotherapy, radiation therapy, breast cancer

## Abstract

Breast cancer is the most commonly diagnosed non-cutaneous cancer and is the leading cause of cancer mortality in females worldwide. Breast cancer incidence has been increasing over the last few decades; simultaneously, novel therapeutic agents including immunotherapies and targeted therapies have become more prominent in use. Radiation therapy continues to serve as a cornerstone to breast cancer treatment in both early-stage and locoregionally advanced disease. Given the improvement in systemic agents, there is increasing interest in investigating the potential synergistic effect of radiation therapy and immunotherapy. As new trials and studies emerge demonstrating the clinical benefits of immune checkpoint inhibitors (ICIs) in breast cancer, especially in PD-L1-positive triple-negative breast cancer (TNBC), it is crucial to investigate the safety and efficacy of combining immunotherapy with radiation treatment. This narrative review discusses the impact of radiation therapy on anti-tumor immunogenicity, and examines the role of immunotherapy and radiation therapy in early-stage, locally advanced, recurrent, and metastatic breast cancer. We conducted a targeted literature search between 2010 and 2024, and included phase II/III clinical trials, mechanistic studies, and ongoing trials that evaluated the combination of immunotherapy (IO) and radiation therapy (RT). We discuss ongoing clinical studies, side effects, and optimal timing of combined IO and RT to enhance therapeutic outcomes.

## 1. Introduction

Breast cancer is the most common non-cutaneous malignancy in women, with an estimated 310,720 new cases of invasive breast cancer diagnosed in the United States in 2024, and 2.3 million new cases globally [1,2,3]. Its incidence has risen over time, particularly among younger women, increasing by approximately 1% annually overall and 1.4% in women under 50 between 2012 and 2021 [4,5]. Despite advancements in detection and treatment, outcomes for patients with high-risk or triple-negative breast cancer (TNBC) remain suboptimal. As novel therapies emerge, integrating immunotherapy (IO) with local treatments such as radiation therapy (RT) has become an area of growing interest.

Radiation therapy is a mainstay of curative-intent treatment for breast cancer. In addition to improving locoregional control, radiation can stimulate immune responses by promoting immunogenic cell death, enhancing antigen presentation, and modifying the tumor microenvironment. These immunologic effects provide a strong rationale for combining RT with immune checkpoint inhibitors (ICIs), which have already transformed outcomes across several tumor types. Although breast cancer has traditionally been considered poorly immunogenic, subtypes such as TNBC and HER2-positive disease exhibit increased immune infiltration and PD-L1 expression. These findings have led to clinical investigation of IO in breast cancer, particularly in combination with RT. Preclinical models and early clinical trials suggest that this combination can enhance anti-tumor immunity and may even elicit systemic responses, such as the abscopal effect.

Investigations are ongoing to determine the optimal sequencing, dosing, and patient selection criteria for immunotherapy in combination with RT in patients with breast cancer. To contextualize these developments and guide future directions, this narrative review aims to provide a timely, comprehensive update of the literature on combining immunotherapy and radiation therapy for breast cancer treatment. Specifically, we first summarize advances in surgical, systemic, and radiation therapies that laid the foundation for current IO–RT strategies. We then review the immunologic rationale for combination therapy, highlight key trials across disease stages, and outline future directions for optimizing safety and efficacy.

We conducted a targeted literature search using PubMed and ClinicalTrials.gov to identify English-language studies published between 2010 and 2024. Search terms included “breast cancer”, “immunotherapy”, “immune checkpoint inhibitors”, “radiation therapy”, and “clinical trial”. We included phase II/III clinical trials, mechanistic studies, and ongoing trials that evaluated the combination of IO and RT. Studies that only individually discussed immunotherapy or radiation therapy, but not the combination, were excluded. This narrative review focuses on preclinical rationale, clinical outcomes, and clinical trials investigating the synergy of immunotherapy and radiation in breast cancer. Additional references were included through manual screening of bibliographies and relevant review articles.

### 1.1. Surgical Advances in Breast Cancer

In 1985, the landmark National Surgical Adjuvant Breast and Bowel Project (NSABP) B-06 trial demonstrated that breast-conserving surgery (BCS) followed by whole breast irradiation (WBI) had equivalent survival outcomes to mastectomy [6]. Breast-conserving therapy (BCT), which consists of partial mastectomy and adjuvant radiation therapy, has since become the standard of care for early-stage breast cancer [7,8]. Adjuvant RT has been shown to reduce both recurrence and breast cancer mortality rates in this setting [9,10].

### 1.2. Developments in Radiation Therapy for Breast Cancer

Radiation therapy has evolved considerably since breast conserving therapy was first introduced nearly 40 years ago. Innovations in technology and clinical research have aimed to optimize dose-fractionation schedules, technique, and sequencing of RT with other modalities.

Initially, conventional fractionation, consisting of 50 Gy in 25 fractions, was the standard dose-fractionation schedule for adjuvant RT. The pursuit of shorter, more convenient regimens led to the development of hypofractionated whole-breast irradiation (HF-WBI), where larger doses per fraction are delivered over a shorter period of time. The pivotal Canadian trial and the UK START A and B trials demonstrated that HF-WBI (40.05–42.56 Gy in 15–16 fractions) provided equivalent local control and cosmetic outcomes compared to conventional fractionation in patients with early-stage breast cancer [11,12,13]. These findings led to international, widespread adoption of HF-WBI. Even shorter dose-fractionation schedules have recently been introduced and adopted. The FAST-Forward trial showed that ultra-hypofractionation (26 Gy in 5 fractions over one week) is a safe and effective alternative to 40 Gy in 15 fractions when delivered in the adjuvant setting for patients with early-stage breast cancer [14].

Parallel to the evolution of WBI, partial breast irradiation (PBI) emerged as an alternative for select low-risk patients, targeting only the lumpectomy cavity with a margin, reducing treatment volume and duration. The RAPID and NSABP B-39/RTOG 0413 trials evaluated external beam and brachytherapy-based PBI, showing comparable local recurrence rates but variable cosmetic outcomes [15,16]. Additionally, the Florence Trial and UK IMPORT LOW trials have shown non inferior recurrence rates in patients with early-stage breast cancer who were treated with partial breast RT as compared to whole breast RT [17,18]. Another innovation, intraoperative radiation therapy (IORT), delivers a single high-dose radiation treatment at the time of surgery, minimizing treatment burden. The TARGIT-A and ELIOT trials demonstrated that IORT is a viable option for select early-stage breast cancer patients, though concerns remain regarding slightly higher recurrence rates compared to WBI [19,20].

Technological advancements have significantly improved the precision and safety of radiation therapy for breast cancer, reducing toxicity while maintaining oncologic efficacy. Intensity-modulated radiation therapy (IMRT) enhances dose conformality for PBI, reducing skin toxicity and improving cosmetic outcomes [21]. Deep inspiration breath-hold (DIBH) has been a major breakthrough for breast cancer, as it reduces cardiac dose and minimizes radiation-induced heart disease [22]. Image-guided radiation therapy (IGRT) has further refined treatment accuracy by allowing real-time imaging, leading to better target coverage and reduced exposure to normal tissues [23,24]. Additionally, proton beam therapy has emerged as a potential option for select patients with challenging anatomy or high long-term cardiac risk including young adults with cancer, with early-phase trials suggesting lower doses to the heart and lungs compared to photon-based radiation [25,26,27].

Collectively, these technological innovations have optimized breast cancer radiation therapy, enhancing both efficacy, patient quality of life, and individualized care. Importantly, understanding the timing and volume differences between approaches such as APBI and hypofractionated RT may guide the optimal sequencing and administration of IO, particularly as the immune microenvironment may vary with RT dose and fractionation. We know that normal tissue versus tumor have different responses and tolerances of radiation therapy; perhaps similarly, the immune environment and response of normal tissues may be dose dependent. Additionally, differences in delivery technique such as proton versus photon RT and their respective impacts on dose distribution to surrounding organs at risk can help guide IO administration and reduce the risk of immune-related toxicity.

### 1.3. The Evolution of Systemic Therapy in Breast Cancer

Chemotherapy has undergone significant evolution in breast cancer treatment, shifting from broad cytotoxic agents to more refined, tailored approaches based on tumor biology [28,29,30,31]. Today, chemotherapy remains a cornerstone in cases of high-risk early-stage, locoregionally advanced, and metastatic breast cancer, often integrated with targeted therapies such as HER2-targeting monoclonal antibodies, immune checkpoint inhibitors [32], and HER2 antibody-drug conjugates including T-DM1 or T-DXd, optimizing efficacy while minimizing toxicity (Table 1).

Hormone therapy soon followed chemotherapy with the discovery that breast cancer can express hormone receptors including estrogen and progesterone. Breast cancer is classified into four molecular subtypes—luminal A, luminal B, HER2-positive, and triple-negative breast cancer (TNBC)—each with distinct characteristics, prognosis, and treatment strategies. Luminal A tumors, characterized by hormone receptor positivity, low Ki-67 levels, and the absence of HER2, have the best prognosis, responding well to endocrine therapy with minimal benefit from chemotherapy [33]. Luminal B tumors, while also hormone receptor-positive, have higher Ki-67 levels, are more proliferative, and derive greater benefit from chemotherapy compared to luminal A [34]. HER2-positive breast cancers, historically associated with poor outcomes, have seen dramatic improvements in oncologic outcomes with targeted therapies such as trastuzumab [35], pertuzumab [36], and T-DM1 [37], which have transformed HER2 overexpression into a therapeutic vulnerability. TNBC, comprising 15–20% of cases, is the most aggressive subtype, lacking hormone receptors and HER2 expression, leading to high early relapse rates and worse overall survival [38]. While chemotherapy remains the mainstay systemic therapy in TNBC, recent advancements, including ICI and antibody-drug conjugates like sacituzumab govitecan [39], offer new hope for improved outcomes in TNBC, particularly in patients with programmed death-ligand 1 (PD-L1)-positive or BRCA-mutated tumors.

This immunologic heterogeneity demonstrates the varying responsiveness of breast cancer molecular subtypes to treatment, highlighting the importance of linking tumor biology with IO efficacy. These insights have laid the foundation for integrating IO with other treatment modalities.

### 1.4. The Evolution of Immunotherapy in Breast Cancer Treatment

Immunotherapy treats cancer by harnessing the body’s immune system to recognize and eliminate malignant cells. Unlike chemotherapy, which targets rapidly dividing cells including tumor cells, immunotherapy enhances immune surveillance and activation through various mechanisms, including immune checkpoint blockade, adoptive cell transfer, and cancer vaccines.

Immune checkpoint blockade works by inhibiting checkpoint proteins, such as cytotoxic T-lymphocyte-associated protein 4 (CTLA-4) and programmed death-1 (PD-1)/PD-L1, which normally act as brakes on the immune response to prevent excessive activation and autoimmunity [40,41]. By blocking these checkpoints with monoclonal antibodies, immune checkpoint blockade restores T cell activity, allowing the immune system to mount a stronger attack against cancer cells. A major breakthrough in oncology was the development of immune checkpoint inhibitors, such as those targeting CTLA-4, PD-1, and PD-L1. The pivotal phase III trial by Hodi et al. (2010) demonstrated that ipilimumab, an anti-CTLA-4 monoclonal antibody, significantly improved overall survival in metastatic melanoma, marking a paradigm shift in cancer immunotherapy [42]. Similarly, the CheckMate-017 and CheckMate-057 trials established nivolumab, an anti-PD-1 agent, as an effective treatment for non-small cell lung cancer (NSCLC) [43,44]. These landmark studies paved the way for immunotherapy’s widespread incorporation, including ongoing investigations into its efficacy in breast cancer, particularly in triple-negative subtypes with high immunogenicity.

Immunotherapy has shown promise in breast cancer, particularly in the treatment of TNBC, which is considered the most immunogenic breast cancer subtype. The KEYNOTE-086 trial evaluated pembrolizumab monotherapy in TNBC and found early evidence of PD-1 blockade efficacy but with modest response rates, leading to the hypothesis that RT could augment immune activation [45,46]. The subsequent phase III KEYNOTE-119 trial found that pembrolizumab did not significantly improve overall survival compared to chemotherapy in previously treated metastatic TNBC, with median survival of 12.7 months versus 11.6 months in patients with PD-L1 CPS ≥ 10 (*p* = 0.057). However, the results suggested potential benefit in PD-L1-enriched subpopulations, highlighting the need for further research on patient selection and combination treatment strategies [47] (Table 1).

The phase III IMpassion130 trial demonstrated the benefit of adding atezolizumab, an anti-PD-L1 monoclonal antibody, to nab-paclitaxel in metastatic TNBC [48]. This combination significantly improved progression-free survival (PFS) but did not show an overall survival benefit, in patients with PD-L1-positive tumors. Similarly, KEYNOTE-355 established pembrolizumab, an anti-PD-1 agent, in combination with chemotherapy as an effective first-line therapy for PD-L1-positive metastatic TNBC [49] (Table 1). Among tumors that expressed PD-L1 with a CPS of 10 or more, the addition of pembrolizumab to chemotherapy resulted in significantly longer overall survival and PFS than chemotherapy alone. Additionally, KEYNOTE-522 demonstrated that adding neoadjuvant pembrolizumab to chemotherapy, followed by adjuvant pembrolizumab, significantly improved pathologic complete response rate (pCR), event free survival (EFS), and overall survival in patients with early-stage TNBC, with a 60-month survival rate of 86.6% versus 81.7% in the placebo-chemotherapy group (*p* = 0.002). The safety profile was consistent with known effects of pembrolizumab and chemotherapy, reinforcing its role as a standard treatment in this setting [32]. However, despite these advances, immunotherapy has shown limited efficacy in hormone receptor-positive (HR+) breast cancers, likely due to their lower tumor mutational burden and immunosuppressive microenvironment [50,51,52].

**Figure 1:**
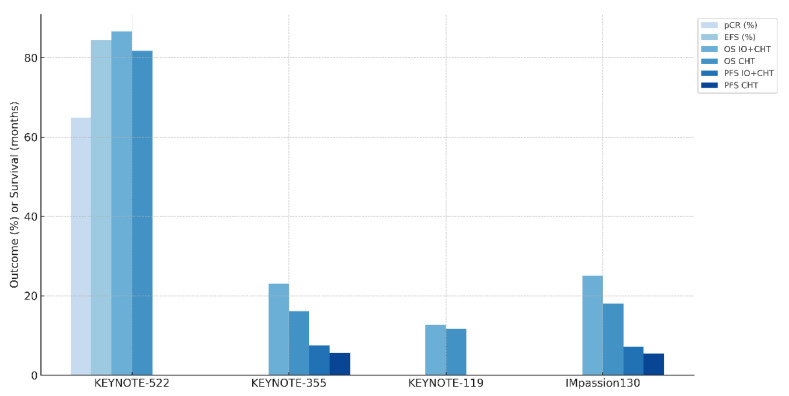
Outcomes from selected multi-institutional, phase III, or above clinical trials for IO in breast cancer. Abbreviations: IO—immunotherapy; CHT—chemotherapy; pCR—pathologic complete response; EFS—event-free survival; OS—overall survival; PFS—progression-free survival.

Figure 1 illustrates key clinical outcomes from selected immunotherapy trials in TNBC. Bars represent pCR, EFS, overall survival (OS), and PFS outcomes where reported. Results are shown for both IO plus chemotherapy (IO + CHT) and chemotherapy (CHT) arms.

Given the limited success of IO alone in breast cancer, researchers have explored combining IO with RT to enhance therapeutic efficacy. The potential synergy between RT and immunotherapy in breast cancer is rooted in the immunomodulatory and immunostimulatory effects of radiation. Radiation can induce immunogenic cell death (ICD), releasing tumor-associated antigens and promoting dendritic cell activation, thereby priming an adaptive immune response [53]. Additionally, RT upregulates major histocompatibility complex (MHC) class I expression, enhancing tumor antigen presentation to cytotoxic T cells [54]. Beyond direct tumor effects, RT can also modulate the tumor microenvironment by depleting immunosuppressive regulatory T cells and myeloid-derived suppressor cells, thereby shifting the immune balance towards an anti-tumor response [55]. Early-phase studies, such as the TONIC trial, have suggested that low-dose radiation may induce an immune-permissive tumor microenvironment, enhancing the response to checkpoint blockade [56]. Furthermore, the abscopal effect—where localized radiation induces systemic tumor regression—has been observed in some patients receiving RT and ICIs, further supporting their synergistic potential [57].

Given these immunostimulatory effects, combining RT with IO represents a promising strategy to overcome the immunosuppressive nature of breast cancer, and ongoing trials aim to determine the optimal sequencing, dosing, and patient selection criteria for this novel therapeutic approach (NCT03199885 [58], NCT04563507 [59], NCT06472583 [60]).

**Table 1:** Selected multi-institutional, phase III or above clinical trials for IO in breast cancer.

	Phase; IO Agent; Enrollment	Disease Stage/ Hormone Receptor Status	Treatment Arms	Primary/ Secondary Endpoints	Treatment Sequence of IO and RT	Study Results and Conclusion or Estimated Completion Date
KEYNOTE-119: Study of Single Agent Pembrolizumab (MK-3475) Versus Single-Agent Chemotherapy for Metastatic Triple Negative Breast Cancer [48]	III; pembrolizumab; 1098 patients	Metastatic TNBC	Pembrolizumab vs. investigator’s choice of single-drug CHT	OS	No radiation therapy within at least two weeks	- Median OS for the overall population: 9.9 months in IO group vs. 10.8 months in CHT group (HR 0.97 [95% CI 0.82–1.15]) - Median OS for patients with a PD-L1 CPS of ≥10: 12.7 months in IO group vs. 11.6 months in CHT group (*p* = 0.057) - Median OS for patients with a PD-L1 CPS of ≥1:10.7 months in IO group vs. 10.2 months in CHT group (*p* = 0.073) No significant OS advantage with pembrolizumab alone over single-agent CHT
KEYNOTE-355: A Randomized, Double-Blind, Phase III Study of Pembrolizumab (MK-3475) Plus Chemotherapy vs. Placebo Plus Chemotherapy for Previously Untreated Locally Recurrent Inoperable or Metastatic Triple-Negative Breast Cancer [45]	III; pembrolizumab; 847 patients	Metastatic or locally recurrent inoperable TNBC	Pembrolizumab + investigator’s choice of CHT vs. placebo + CHT	OS, PFS, Percentage of patients with AE and those who discontinued study drug due to an AE	N/a	- Median OS for the overall population: 17.2 months in IO + CHT group and 15.5 months in placebo + CHT group (significance not tested) - Median OS for patients with a PD-L1 CPS of ≥10: 23.0 months in IO + CHT group vs. 16.1 months in placebo + CHT group (*p* = 0.0185) - Median OS for patients with a PD-L1 CPS of ≥1:17.6 months in IO + CHT group vs. 16.0 months in placebo + CHT group (not significant) - AE of grade 3–5: 68.1% in IO + CHT group vs. 66.9% in placebo + CHT group Significant OS advantage with pembrolizumab + CHT compared to CHT alone for patients with advanced TNBC with PD-L1 CPS of ≥10
KEYNOTE-522: Study of Pembrolizumab (MK-3475) Plus Chemotherapy vs. Placebo Plus Chemotherapy as Neoadjuvant Therapy and Pembrolizumab vs. Placebo as Adjuvant Therapy in Participants With Triple-Negative Breast Cancer [32,61,62]	III; pembrolizumab; 1174 patients	Previously untreated stage II-III TNBC	Pembrolizumab + CHT (paclitaxel and carboplatin) vs. placebo + CHT	pCR; EFS	No radiation therapy within the past 12 months	- pCR (of the first 602 patients who underwent randomization): 64.8% in IO + CHT group vs. 51.2% in placebo + CHT group (*p* < 0.001) - EFS at 36 months: 84.5% in IO + CHT group vs. 76.8% in placebo + CHT group (statistically significant) - (Secondary endpoint) OS at 60 months: 86.6% in IO + CHT group vs. 81.7%% in placebo + CHT group (*p* = 0.002) Significant OS advantage with pembrolizumab + CHT compared to CHT alone
IMpassion130: A Study of Atezolizumab in Combination With Nab-Paclitaxel Compared With Placebo With Nab-Paclitaxel for Participants With Previously Untreated Metastatic Triple-Negative Breast Cancer [31,63]	III; atezolizumab; 902 patients	Previously untreated metastatic TNBC	Atezolizumab + nab-paclitaxel vs. placebo + nab-paclitaxel	PFS; OS	N/a	- Median PFS: 7.2 months in IO + CHT group vs. 5.5 months in placebo + CHT group (*p* = 0.002) - Median OS: 21.0 months in IO + CHT group vs. 18.7 months in placebo + CHT group (*p* = 0.078) - (Exploratory analysis) median OS in patients with PD-L1 immune cell positive tumors: 25.0 months in IO + CHT group vs. 18.0 months in placebo + CHT group (HR 0·71 [95% CI 0·54–0·94]) No significant OS advantage with the addition of atezolizumab in the overall population, but perhaps a clinically meaningful OS advantage for patients with PD-L1 immune cell-positive tumors

Abbreviations: AE—adverse event, CHT—chemotherapy, CI—confidence interval, EFS—event-free survival, HR—hazard ratio, IO—immunotherapy, N/a—not available, OS—overall survival, pCR—pathologic complete response, PFS—progression-free survival.

## 2. Breast Cancer Immune Microenvironment

The immune microenvironment in breast cancer is a dynamic and complex ecosystem composed of various immune cell populations that can exert both pro-tumorigenic and anti-tumorigenic effects. In order to proliferate and metastasize, cancer cells must first escape eradication by the immune system [64]. Avoidance of recognition by the immune system is thought to be due to various mechanisms, including downregulation or inactivation of receptors that facilitate an immune response, such as MHC class I-related machinery. Simultaneously, cancer cells promote an immunosuppressive microenvironment, facilitating its growth. Cancer cell proliferation recruits regulatory T cells and myeloid-derived suppressor cells, which produce anti-inflammatory cytokines such as Transforming Growth Factor-Beta (TGF-β) and interleukin (IL)-10, resulting in inhibition of cytotoxic T cells [65,66]. Additionally, cytotoxic T cells can be destroyed by tumor vascular endothelium through engagement of PD-1 receptor and expressing PDl1 ligand [65].

Tumor-infiltrating lymphocytes (TILs), particularly cytotoxic CD8^+^ T cells, play a crucial role in anti-tumor immunity by recognizing and eliminating malignant cells through antigen presentation and direct cytotoxic activity [67]. High TIL levels have been associated with improved prognosis and enhanced responses to therapies, particularly in triple-negative and HER2-positive breast cancers [68]. A pivotal study by Loi et al. demonstrated that in TNBC, each 10% increase in intratumoral and stromal lymphocytic infiltrations was associated with a 15% reduction in risk of recurrence and 17% reduction in death among patients receiving chemotherapy [69]. Conversely, low TILs are associated with poorer outcomes and commonly seen in patients with older ages, larger tumor sizes and lymph node metastases [70]. Based on this data, TIL is suggested to be used as a prognostic biomarker in TNBC and HER2-positive breast cancers [71].

Immune infiltration in breast cancer varies significantly by molecular subtype, with important implications for stratification in IO-RT clinical trials. Because TNBC exhibits the highest immunogenicity, with elevated TILs in 2–30% of cases and PD-L1 expression in up to 60%, this subtype is more likely to respond to IO. HER2-positive tumors exhibit intermediate levels of immune infiltration and may benefit from dual HER2- targeted and IO treatments. In contrast, hormone receptor-positive, HER2-negative breast cancers are the most common subtype, and are associated with low immunogenicity due to low TILs and low PD-L1 expression (<10%), consistent with their decreased response to IO [72].

However, the breast tumor microenvironment is also rich in immunosuppressive components, including regulatory T cells (T regs) and myeloid-derived suppressor cells (MDSCs), which dampen the anti-tumor response and facilitate immune evasion [73]. Tumor-associated macrophages (TAMs), which can polarize into either pro-inflammatory (M1) or immunosuppressive (M2) phenotypes, further influence tumor progression; while M1 macrophages promote an anti-tumor response through cytokine secretion and antigen presentation, M2 macrophages support tumor growth, angiogenesis, and metastasis [74]. Breast cancer uniquely exhibits an immune microenvironment that varies by molecular subtype, with TNBC generally displaying higher immune infiltration and immunogenicity compared to luminal subtypes, which are less responsive to immune checkpoint blockade.

Ultimately, re-establishing immune recognition of tumors as foreign and augmenting pre-existing immunity are the crux of immunotherapeutic agents in attempt to destroy these immune-evading cancer cells. Understanding the dynamic interplay between immune cells in the breast tumor microenvironment is critical for developing novel immunotherapeutic strategies to overcome immune suppression and enhance anti-tumor immunity.

## 3. Mechanism and Rationale for Use of Immunotherapy and Radiation Therapy

Radiation triggers immunosuppression by ionizing cells’ DNA leading to DNA damage and cell death. This is done through various interactions that are described below. It is believed that radiation therapy in combination with immunotherapy may work synergistically through priming tumor-specific cytotoxic T cells, promoting the function of these T cells, and blunting the tumor’s immunosuppressive microenvironment. These mechanisms have been demonstrated in preclinical models; however, direct evidence supporting these interactions in clinical breast cancer remains limited. The proposed mechanisms are outlined in the sections below.

### 3.1. Immunogenic Cell Death and DAMP Signaling

**Figure 2:**
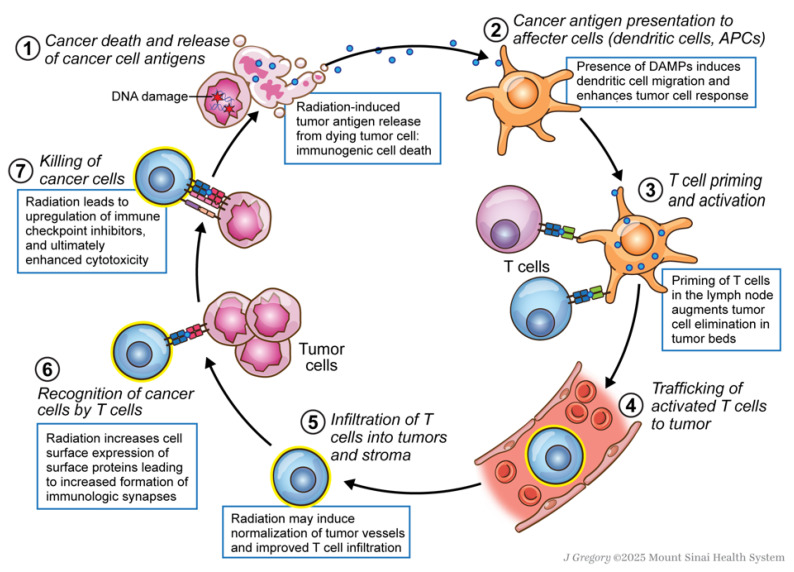
The impact of radiation therapy on antitumor immunity across the cancer–immunity cycle. Radiation induces DNA double-strand breaks resulting in cell death and release of tumor antigens, a process known as immunogenic cell death [75,76]. The resulting DNA damage leads to the release of DAMPs, which promote dendritic cell recruitment and activation [77]. These dendritic cells prime T cells in the lymph nodes, augmenting cytotoxic T cell-mediated tumor elimination [75,78]. T cells are recruited to the tumor bed through this priming by APCs and through the action of chemokines and other signals released by the tumor microenvironment. At the site of the tumor, radiation normalizes tumor vasculature, improving T cell infiltration into the tumor microenvironment [79]. Radiation also increases cell surface expression of proteins such as MHC class I and other surface proteins, enhancing immunologic synapse formation between T cells and cancer cells [54]. Finally, radiation upregulates immune checkpoint inhibitors such as PD-1/PD-L1, sensitizing tumors to immune checkpoint blockade and further enhancing cytotoxic T cell responses [80]. Abbreviations: APC—antigen-presenting cell; DAMP—damage-associated molecular patterns; DNA—deoxyribonucleic acid; MHC—major histocompatibility complex; PD-1/PD-L1—programmed cell death protein 1/programmed death-ligand 1.

Combining radiation therapy with immunotherapy is believed to be advantageous as radiation therapy mobilizes an anti-tumor immune response. Immune checkpoint inhibitors target proteins within the cell cycle to inhibit apoptosis of T cells, which allows for T cells to evade immune destruction. The increased quantity and efficacy of T cells allow for a more robust anti-tumor response. Radiotherapy causes damage in the DNA of cancer cells primarily through the production of DNA double-stranded breaks. As a result, the tumor cell dies, and releases antigens resulting in recruitment of the individual’s immune system, termed immunogenic cell death [75,76]. DNA damage from RT is thought to result in the release of tumor neoantigens, specifically damage-associated molecular patterns (DAMPs) [77], see Figure 2. DAMPs incite recruitment and activation of antigen-presenting cells, and promote downstream particles such as adenosine triphosphate (ATP), calreticulin, high mobility group box 1 (HMGB1) and type I interferon (IFN-1) that attract immune cells, particularly dendritic cells, to the site of death.

### 3.2. Activation of the STING Pathway

The presence of DAMPs in the cytoplasm activates the cGAS/STING pathway to signal for increased production of IFN-1 which induces dendritic cell migration to the tumor and cross-priming of T cells. DAMPs also promote the expression of calreticulin on the cell surface, which promotes phagocytosis by dendritic cells, as well as HMGB1 which is an agonist for toll-like receptors on the dendritic cell surface to induce an antigen specific T cell response [67]. Additionally, recent studies have demonstrated the role of inflammatory monocytes and macrophages in stimulating intra-tumoral T cells. These monoctyes present peptide-MHC class I complexes from tumor cells to T cells, further contributing to immune regulation [81]. Other immune signaling pathways, including the RIG-1 pathway to sense cytoplasmic RNA, and NK-cell-based pathways, have also been shown to be induced by radiation therapy [82]. Some preclinical breast models suggest radiation therapy induces neoantigen presentation not only in tumor cells, but also in tumor draining lymph nodes, promoting CD8+ T cell mediated cytotoxicity [72]. However, clinical evidence in breast cancer remains preliminary. Thus, in addition to the direct cellular death due to the localized DNA damage of tumor cells, radiotherapy may also result in indirect cellular death as a result of this immunomodulation.

### 3.3. Upregulation of Immune Checkpoints

Immunogenic cell death also involves upregulation of PD-L1, which inhibits T cell production and activity. DNA double-strand breaks have been shown to upregulate PD-L1, providing insight into how RT enhances PD-L1 expression on tumor cells. Moreover, the depletion of DNA repair proteins in response to radiotherapy has been shown to enhance PD-L1 induction [80]. The use of RT in combination with ICI allows for potential synergistic enhancement of the cytotoxic effects of PD-L1 inhibitors through increasing PD-L1 expression and enhancing PD-L1 induction, thereby minimizing the tumor’s immunosuppressive effects.

Immunotherapy targets PD-L1 and CTLA-4 allow for a synergistic effect with RT to potentially improve clinical outcomes. Additionally, radiation therapy plays an important role in the tumor microenvironment. Radiation therapy may affect tumor vasculature including promoting an adhesive phenotype, improving transmigration of immune effector cells [79]. As to the interaction between RT and antibody interactions, a study by Demaria et al. investigated RT in combination with a CTLA-4 antibody in a mouse model with metastatic mammary carcinoma and found that the combination of RT and CTLA-4 antibody delayed metastases and improved overall survival [83]. CTLA-4 is an inhibitory receptor that is up-regulated when naive T cells are stimulated to suppress T cell activation and proliferation. Another pre-clinical study in breast murine models by Deng et al. demonstrated the upregulation of PD-L1 after RT and a subsequent amplification of antitumor response when RT was combined with PD-L1 blockade [84]. These preclinical studies provide the rationale for further research in human studies on the use of RT and immunotherapy in breast cancer.

Finally, there is evidence that there is a systemic antitumor immune response induced by radiation, as demonstrated by shrinkage of tumors outside of the radiation field in the setting of radiotherapy monotherapy in select cases: this is called the abscopal effect. What was previously a rare phenomenon is now increasingly reported in settings in which patients were treated with radiotherapy due to progression on immunotherapy. This phenomenon is thought to be due to priming of T-cells in lymph nodes, and improved recognition and killing in the irradiated tumor bed, as well as other distant sites [75,78].

## 4. Immunotherapy and Radiation Therapy for Early-Stage and Locally Advanced Breast Cancer

The integration of RT with IO is an evolving strategy in the treatment of early-stage and locally advanced breast cancer, particularly in TNBC, where treatment options remain limited. Radiation not only provides local tumor control but also has immunomodulatory effects that may enhance the efficacy of immune checkpoint inhibitors, as explained above. Recent clinical trials have explored the feasibility of combining RT with IO in both the neoadjuvant and adjuvant settings, with promising results in improving pathological response rates and early survival outcomes. However, challenges such as treatment toxicity and patient selection remain critical considerations, particularly in the post-neoadjuvant setting. This section examines key clinical trials investigating the role of immunotherapy and radiation in early-stage and locally advanced breast cancer, highlighting potential benefits, limitations, and future directions.

One study investigating this approach explored the combination of a single 7 Gy RT fraction with a single preoperative pembrolizumab dose in patients with cT1N0 TNBC (NCT04454528). Given that standard neoadjuvant chemo-immunotherapy may be excessive for early-stage TNBC, this phase Ib/II trial aimed to evaluate the feasibility of a de-escalated IO regimen. The study design involved three neoadjvuant treatment arms, where patients received either RT followed by pembrolizumab, pembrolizumab followed by RT, or pembrolizumab alone, with feasibility and tolerability as primary endpoints. Secondary endpoints included changes in tumor-infiltrating lymphocytes (TILs) and pathologic response rates. The results demonstrated a major pathologic response (<10% residual invasive tumor) in 33% of TNBC patients who received neoadjvuant Pembrolizumab and RT, with one achieving a complete response. Additionally, TNBC tumors in all arms showed a significant increase in TILs post-treatment, suggesting enhanced immune activation [85]. It is important to note the small sample size and lack of long-term survival data limit the generalizability of this study. However, these findings do highlight the potential of RT combined with immune checkpoint blockade to induce effective tumor immune responses, laying the groundwork for further studies into de-escalated preoperative immunotherapy strategies for early-stage TNBC.

Building upon the role of checkpoint inhibitors in early-stage TNBC, the KEYNOTE-522 trial, a phase 3 randomized study, evaluated the impact of adding pembrolizumab to neoadjuvant chemotherapy in patients with Stage II or III TNBC (Table 1). Patients were randomized to receive either pembrolizumab plus paclitaxel/carboplatin followed by anthracycline-based chemotherapy or chemotherapy alone, with both arms continuing adjuvant pembrolizumab or placebo post-surgery whilst receiving adjuvant radiation treatment. No details of the adjuvant radiation treatment were provided. The primary analysis demonstrated a significantly higher pCR rate in the pembrolizumab group (64.8%) compared to chemotherapy alone (51.2%). With a median follow-up of 15.5 months, pembrolizumab also improved EFS (HR: 0.63), although long-term survival benefits remain under investigation. The incidence of grade 3 or higher AEs was slightly elevated in the pembrolizumab group (78% vs. 73%), yet immune-related toxicities were manageable [86]. These results reinforce pembrolizumab as a key component of neoadjuvant therapy in high-risk early-stage TNBC, demonstrating enhanced tumor response and improved early survival outcomes. These results further support the safety of IO when given in conjunction with adjuvant RT to the breast. The concurrent use of chemotherapy makes it difficult to isolate the IO effect, and the unknown timing or dosing of adjuvant radiation treatment are important limitations in this study.

Additional studies are investigating the combination of immunotherapy and radiation treatment together in the neoadjuvant setting. An ongoing phase I/II trial is investigating the feasibility of neoadjuvant pembrolizumab with combined RT boost to the primary breast tumor followed by standard breast conservation therapy (breast-conserving surgery and adjuvant RT to the whole breast) and chemotherapy. In this study, high-risk patients defined as patients with ER-positive disease, HER2-negative with 2 of the following criteria: histologic grade 2–3, Ki-67 > 20%, ER expression <75%; or patients with TNBC, will receive two neoadjuvant doses of pembrolizumab, the second dose delivered with a radiation “tumor boost”, 24 Gy in 3 fractions (NCT03366844 [87]). The primary outcome includes change in TILs 8 weeks after trial initiation, with an increase in TILs indicating immune system engagement with the hypothesis that this will correspond with a better clinical outcome. This study provides a basis of using TIL levels pre and post treatment as a predictor for IO response and prognostic factor.

While neoadjuvant checkpoint blockade has demonstrated promise, its role in the post-neoadjuvant setting remains uncertain, as evidenced by the BreastImmune-03 trial, a phase II study evaluating nivolumab (anti-PD-1) plus ipilimumab (anti-CTLA-4) versus capecitabine in TNBC patients with residual cancer burden after neoadjuvant chemotherapy and surgery. This trial aimed to determine whether a dual immunotherapy regimen could improve DFS and OS compared to capecitabine, a historical standard for this high-risk population. The study was prematurely terminated due to an unexpectedly high incidence of myocarditis (11%) in the IO arm, leading to concerns regarding safety. With a median follow-up of 34.3 months, there was no significant improvement in DFS (HR: 0.84) or OS (HR: 0.98) in the nivolumab/ipilimumab group. Furthermore, 38% of patients discontinued IO due to treatment-related AEs, compared to only 14% in the capecitabine arm. These treatment related AEs included hypothyroidism, adrenal insufficiency, diabetes mellitus, colitis, hepatitis, polymyalgia rheumatica, and pneumonitis [88]. These findings suggest that nivolumab/ipilimumab does not provide meaningful survival benefits in this setting, and the high toxicity profile limits its clinical safety. However, ongoing translational studies may identify biomarkers to refine patient selection for future dual immunotherapy strategies.

Furthermore, a study by Tison et al. investigated the potential role and tolerance profile of adjuvant radiation with concurrent pembrolizumab in patients with early and locally advanced TNBC who received neoadjuvant chemo-immunotherapy with pembrolizumab. Fifty-five patients were included, 28 of which received adjuvant RT with concurrent pembrolizumab, and 27 patients who received adjuvant RT only. The study did not find a difference in the toxicity between adjuvant RT with versus without concurrent IO [61].

Collectively, these studies underscore the emerging role of immunotherapy and radiation in early-stage and locally advanced breast cancer. The combination of RT with checkpoint blockade may represent a potential de-escalation strategy in select early-stage TNBC cases, particularly those with favorable immune microenvironments, such as high TILs. For example, as evidenced by the preliminary efficacy seen in NCT04454528, suggests that a short course RT and IO regimen can induce pathological responses without systemic therapy [85]. Meanwhile, neoadjuvant immunotherapy with chemotherapy, as demonstrated in KEYNOTE-522, has become a common approach for Stage II and Stage III TNBC, improving pCR rates and early EFS [86] (Table 1). Additional studies have highlighted challenges associated with the use of IO, for example BreastImmune-03 trial highlights the potential challenges of checkpoint blockade in the post-neoadjuvant setting, where toxicity concerns outweigh clinical benefits at this time [89]. These findings emphasize the need for continued research into patient selection, optimal sequencing, and novel RT-IO combinations to maximize therapeutic efficacy while minimizing toxicity in early-stage and locally advanced breast cancer.

## 5. Immunotherapy and Radiation Therapy in the Recurrent and Metastatic Setting

Although advances in treatment modalities have led to improved survival for patients with breast cancer, metastatic breast cancer remains incurable and is the second leading cause of cancer death among women [90]. Current treatment options for metastatic or recurrent breast cancer include surgery, radiation, chemotherapy, endocrine therapy, targeted biological therapy, and immunotherapy.

There are a limited number of clinical trials that are or have investigated the combination of immunotherapy and radiation in metastatic breast cancer. An early phase I study by Luke J. et al. evaluated the safety of pembrolizumab with multisite stereotactic body radiation therapy (SBRT) in 73 patients with metastatic solid tumors, including ovarian/fallopian tube cancer, non-small cell lung cancer, breast cancer, cholangiocarcinoma, endometrial cancer, colorectal cancer, and head and neck cancer. SBRT dosing varied by site and ranged from 30 to 50 Gy in three to five fractions. Most of these patients (94.5%) received SBRT to two metastatic lesions. Six of these patients included women with metastatic breast cancer. The overall response rate of the entire cohort was 13.2%. Although this study did not include overall response rates based on histology of metastatic/primary lesions, it suggests a possible synergistic response between RT and IO, laying the groundwork for future clinical trials [91].

A recently completed pilot study investigated the safety and biological effects of combining SBRT and pembrolizumab in 15 patients with oligometastatic breast cancer (NCT02303366 [62]). Enrolled patients were treated with SBRT delivered to a dose of 20 Gy in 1 fraction to oligometastatic disease and pembrolizumab for 6 months, 1 dose every 3 weeks for a total of 8 cycles. The investigators hypothesized evidence of systemic immune activation with the combination of treatments; final results are pending at this time.

There are ongoing trials investigating the role of radiation and immunotherapy for metastatic breast cancer with at least 2 brain metastases (NCT03449238 [63]). In this trial, patients will complete stereotactic radiosurgery (SRS) to a standard dose and receive pembrolizumab infusion on day 4 and repeated every 3 weeks until evidence of brain progression or adverse toxicity. The estimated primary trial completion date is December, 2025.

### 5.1. Metastatic TNBC

Metastatic triple negative breast cancer is associated with a poor prognosis and limited treatment options [92]. However, prior studies demonstrate robust tumor T cell infiltrate and increased expression of PD-L1 in patients with TNBC, hypothesized to be due to increased chromosomal instability and mutations [93,94]. This suggests that metastatic TNBC may be more sensitive to IO and RT treatment. A phase II clinical trial investigated the efficacy and safety of pembrolizumab and RT in 17 patients with metastatic TNBC who were unselected for PD-L1 expression. Patients received SBRT to metastatic lesions, delivered with a dose of 30 Gy in 5 fractions and pembrolizumab was administered within three days after the first fraction of radiation. The overall response rate was 17.6% (95% CI, 4.7–44.2%), with 3 complete responses, 1 case of stable disease, and 13 cases of progressive disease. Most common grade 1 to 2 toxicity was dermatitis (29%). Overall, these results suggest that pembrolizumab and RT for metastatic TNBC is without significant toxicity and may be associated with improved response rate [95].

The phase II TONIC trial sought to evaluate immune induction strategies to enhance sensitivity to PD-L1 blockade in patients with metastatic TNBC. Patients (n = 67) were randomized to nivolumab without induction or with either short course of induction therapy with cyclophosphamide, cisplatin, doxorubicin, or RT to a single metastatic lesion to a dose of 24 Gy in 3 fractions followed by nivolumab. The overall response rate was 20%. The response rates were doxorubicin—35%; cisplatin—23%; radiation—8%; cyclophosphamide—8%; no induction therapy—17%. These findings suggest that short-term chemotherapies such as doxorubicin and cisplatin may induce a favorable tumor microenvironment and increase response to PD-1 blockade. Of note, radiation led to a worse response rate than no induction therapy, although this is a non-comparative phase II trial. This unexpected result suggests that RT used in this setting was likely not able to induce a favorable immune microenvironment. Possible explanations for this include the dose, fractionation, or timing between RT and IO. In the future, it would be of interest to compare this chemo-induction strategy to one with and without radiation therapy to see if RT would further augment the tumor response in the appropriate setting, or to increase the dose/fractionation to a conventional breast treatment regimen [56].

### 5.2. Hormone Receptor-Positive Metastatic Breast Cancer

Despite being the most common breast cancer subtype, hormone receptor-positive, HER2-negative breast cancer has shown minimal benefit from IO, likely due its immunologically “cold” phenotype, as evidenced by low levels of TILs and PD-L1 expression (<10%) [72]. However, as hypothesized above, radiation therapy has the potential to convert “cold” tumors into IO responsive tumors by increasing antigen presentation, upregulating type I interferon signaling, promoting chemokine release, and enhancing T- cell infiltration. A phase II trial included eight patients with HR-positive and HER2-negative metastatic breast cancer who were candidates for palliative radiation to 20 Gy in 4 fractions to previously unirradiated sites. Patients received pembrolizumab two to seven days prior to radiation. There were no objective responses observed in the first eight patients, and the trial was subsequently closed to accrual [96].

Future studies investigating the use of biomarkers to predict response to IO and refine patient selection are currently underway. PD-L1 expression and combined positive score are currently the most commonly used biomarkers in clinical trials. However, their predictive value in breast cancer remains inconsistent due to the presence of different antibodies in breast cancer subtypes, lack of interchangeability between assays, and variability in combined positive scoring systems. Additionally, malignancies with higher levels of mutations, or tumor mutational burden, have been reported to be involved in a more robust immune response. Recent studies have demonstrated that approximately 10% of breast cancers express high tumor mutational burden, and may have a potential association to response to IO. However, further studies are required to create a standardized method to assess and use tumor mutational burden as a biomarker in breast cancer [97].

## 6. Immunotherapy and Radiation Therapy and Other Systemic Agents

As previously discussed, immunotherapy combined with radiation therapy can augment immunological effects, leading to an increase in locoregional control. Combining immunotherapy, radiation, and other targeted or systemic therapies may also be a promising means for improving response rates.

### 6.1. Poly (ADP-Ribose) Polymerase

Prior studies have hypothesized that combining poly (ADP-ribose) polymerase (PARP) inhibitors with radiation therapy can inhibit DNA repair functions, thereby increasing the efficacy of radiation [98]. PARP inhibitors impair the repair of single-strand DNA breaks. When combined with radiation, which induces double-strand breaks, tumor cells become more susceptible to lethal damage due to the simultaneous accumulation of unrepaired DNA and compromised repair mechanisms. PARP inhibitors and RT have been shown to modulate inflammatory signals by enhancing the infiltration of cytotoxic T lymphocytes into the tumor bed, stimulating an immune response. Furthermore, PARP inhibitors and RT have been shown to upregulate PD-L1 expression, increasing tumor sensitivity to immunotherapy [99]. There are several ongoing clinical trials investigating the response rate to combined IO, PARP inhibitors, and radiation treatment. A phase II trial is investigating the effects of niraparib—a PARP inhibitor, dostarlimab—PD-L1 checkpoint inhibitor, and SBRT to 24 Gy in 3 fractions in patients with PD-L1 positive metastatic TNBC who have progressed on prior immunotherapy (NCT04837209 [100]). Similarly, another phase II trial is studying the therapeutic effects of atezolizumab—a PD-L1 inhibitor, talazoparib—a PARP inhibitor, and SBRT to 24 Gy in 3 fractions to one to four metastatic lesions in patients with BRCA 1/2 negative patients with PD-L1-positive metastatic TNBC (NCT04690855 [101]). A final study is investigating the effects of the PARP inhibitor olaparib in combination with ICI or RT in patients with metastatic TNBC. Patients will be randomized to either receive SBRT to 24 to 27 Gy in 3 fractions and pembrolizumab, with or without olaparib (NCT04683679 [102]).

Despite these encouraging approaches, overlapping toxicities may limit the combination of IO, PARP inhibitor, and RT. These toxicities may include hematologic and gastrointestinal side effects. Furthermore, it is currently unclear which patients would derive the most benefit from combination therapy; further investigation into the integration of biomarkers is required to aid in patient stratification.

### 6.2. Nanoparticle Radioenhancers

NBTXR3 is a novel radioenhancer composed of hafnium oxide nanoparticles designed to augment the local effects of radiotherapy. When activated by ionizing RT, NBTXR3 increases tumor cell death and may potentiate systemic immune responses. A phase I clinical trial (NCT03589339 [103]) is evaluating NBTXR3 in combination with anti-PD-1 IO and RT in patients with advanced cancers, including breast cancer. This approach aims to enhance local tumor control and stimulate systemic antitumor immunity.

## 7. Tolerability and Toxicities

As aforementioned, it is believed that the addition of immunotherapy to radiation and vice versa augments the anti-tumor immune response. However, a stronger immune response may also be a concern for increased toxicity due to potential overactivation of immunogenic effects against healthy tissue. For this reason, several studies have been conducted to evaluate the feasibility and tolerability of radiation with immunotherapy.

Toxicity associated with breast RT involves dermatitis, edema, fatigue, and pain/tenderness acutely. Within weeks to months post-treatment, patients may develop subacute toxicities which may include pneumonitis or fat necrosis, and late toxicities including fibrosis, breast edema, lymphedema, chest wall/rib pain and fracture, cardiotoxicity, and secondary malignancies [104,105,106]. The use of IO in the clinical setting is associated with a side effect profile due to dysregulation of the immune system, coined immune-related adverse events (irAEs) [107]. Immune-related AEs can affect any organ system and manifest as an inflammatory reaction of the involved tissue. For example, dermatologic irAEs include rash, pruritus, dermatitis, and vitiligo, while pulmonary irAEs include pneumonitis and sarcoidosis [108]. Because both RT and IO have reported toxicities associated with each treatment, it is crucial to assess the toxicity profile and feasibility of the combined use of IO and radiation therapy.

The overlapping toxicities of immunotherapy and radiotherapy are multifactorial and stem from synergistic effects on immune activation and tissue inflammation. Immune-related AEs arise from checkpoint inhibitor-induced dysregulation of immune tolerance, leading to T cell infiltration and inflammation in healthy tissues. Common irAEs include dermatitis, colitis, pneumonitis, and endocrinopathies, with pneumonitis being of particular concern in thoracic radiation settings. Radiotherapy itself can independently induce inflammation and tissue damage via direct DNA injury, reactive oxygen species generation, and cytokine release, which can be amplified when immune activation is concurrently enhanced by checkpoint blockade [93,94,95]. This potentiation may increase the risk and severity of tissue-specific toxicities, such as pneumonitis, radionecrosis, or fibrosis. There is limited data on the safety of immunotherapy and radiation therapy in breast cancer, but prior studies have indicated tolerability of the combination of immunotherapy and RT in other disease areas. A secondary analysis of KEYNOTE-001 phase 1 trial investigated patients with non-small cell lung cancer who previously underwent RT before receiving pembrolizumab. This study sought to assess whether previous RT affected toxicity and survival outcomes. A total of 98 patients were enrolled in the trial, and 42 of these patients had received RT and 24 of these patients had received thoracic RT. A total of 63% of the 24 patients treated with thoracic RT reported pulmonary toxicity versus 40% of the 73 patients who had no prior thoracic RT. This study suggests that the combination of IO with prior RT has an acceptable safety profile [109]. Similarly, another study evaluated the safety of lung SBRT with concurrent immune checkpoint inhibition. A total of 117 patients were included, and 54 of these patients received SBRT with concurrent ICI and 63 patients received SBRT alone. The results of this study demonstrated a higher risk of grade 3 pneumonitis in patients who were treated with SBRT and ICI versus SBRT alone (10.7% vs. 0%). However, the risk of any grade pneumonitis was not statistically significant between the two groups. The results of this study demonstrate the overall safety of the combined use of SBRT and ICI; however, the authors do recommend closer monitoring for patients receiving RT with ICI [110].

Furthermore, a retrospective review analyzed the rate of radionecrosis in 137 patients with stage IV melanoma who were treated with the combination of RT, ipilimumab, and/or pembrolizumab. A total of 1094 metastatic brain lesions were treated with radiation therapy. A total of 87% of these patients received ipilimumab, 9% received pembrolizumab, and 4% received both within 1 year of radiation. The rate of radionecrosis was 27% and was associated with a larger size and number of lesions treated [111].

Similarly, another retrospective study analyzed 17 patients with 49 brain metastases with lung primaries who were treated with SRS before (22 lesions), during (13 lesions), or after IO (14 lesions). The study found no increased toxicity with RT and immunotherapy. Interestingly, this study found the combination of RT used in close proximity with IO improved distant disease control [112].

However, the data is conflicting, and combination therapy has also been associated with increased toxicity. Prior studies have demonstrated this toxicity to be associated with prior comorbidities, radiation dose, and temporal proximity of RT and immunotherapy. In the prospective phase 3 PACIFIC trial, patients with non-small cell lung cancer were treated with sequential chemoradiation followed by either durvalumab or placebo. This study reported radiation pneumonitis in 34% of patients treated with durvalumab versus 25% of patients who received the placebo [113]. This study also underscores that even sequential treatment can result in increased toxicity. Additionally, a review by Azhar et al. demonstrated increased risk of radiation-induced lung injury and ICI-pneumonitis in patients with pre-existing interstitial lung disease [114]. Another retrospective review analyzed 41 patients with a history of irAEs that were subsequently treated with thoracic RT, and found these patients to be at a very high risk for radiation pneumonitis. This toxicity has been shown to have a strong dose–response relationship between mean lung radiation dose [109]. These studies demonstrate the importance of considering patient comorbidities, radiation technique and dose, and timing between RT and IO in order to limit toxicity. When RT and IO are combined, overlapping toxicities can result from their shared pro-inflammatory pathways like IFN-γ, TNF-α, and IL-6, and may lead to increased expression of PD-L1 in irradiated tissues, fueling a feedback loop of immune activation [71,74]. This can increase the risk of radiation pneumonitis, radionecrosis, or fibrosis. Clinically, management requires early recognition of overlapping toxicities and multidisciplinary coordination. Corticosteroids remain the cornerstone for moderate-to-severe irAEs, with treatment interruption or discontinuation depending on severity. There is no clear consensus regarding the efficacy and toxicity profile of concurrent vs. sequential IO and RT.

The tolerability and potential toxicity of combination immunotherapy and radiation therapy for treatment of breast cancer continues to be explored. Table 2 summarizes the toxicities reported in key clinical trials for IO in breast cancer.

In the KEYNOTE-522 trial, neoadjuvant chemotherapy with pembrolizumab for patients in TNBC with increased PD-L1 expression demonstrated improved pathologic complete response rates (Table 1). It was noted that the pembrolizumab group had higher rates of AEs, including severe skin reactions (3.8%), infusion reactions (2.6%), and adrenal insufficiency (1.3%), but most AEs occurred during the neoadjuvant phase of treatment [86]. In the IMpassion130 trial, the atezolizumab–nab-paclitaxel group showed increased grade 3 and 4 toxicity compared to the placebo–nab-paclitaxel group, particularly peripheral neuropathy, though RT toxicity is not discussed [48] (Table 1). Data regarding the sequencing of RT with IO is limited. Optimal sequencing of RT and IO is an important factor in patient outcomes. For instance, one retrospective review analyzed the impact of timing of IO and SRS in 75 melanoma patients with 566 brain metastases. The study showed an overall survival benefit and reduction in lesion volume with concurrent SRS and ICI compared to sequential (>4 weeks apart) [115].

The sequencing of immunotherapy with breast radiotherapy has been challenging to evaluate as many of the studies discussed above do not describe their radiation methodology, dosing, fractionation, or timing. A systematic review conducted to delineate treatment details, safety, and efficacy in patients with breast cancer showed that there was high variability in the dosing, fractionation, technique, treatment volumes, and timing of radiation between patients within studies examining patients with breast cancer [86,112,115]. Of note, a post hoc exploratory subgroup analysis of phase 3 KEYNOTE-522 compared EFS, stratifying for subgroups of patients without adjuvant RT, with adjuvant RT, sequential RT, and concurrent RT (Table 1). Six percent of patients who received pembrolizumab with adjuvant RT were reported to have grade 3–5 AEs, as compared to 2.7% of patients in the placebo and RT arm. Two of the patients (0.4%) in the pembrolizumab and adjuvant RT arm were reported to have a treatment-related AE that led to death. The most common AEs were nausea, alopecia, and anemia. This study demonstrates the safety and tolerability of both regimens [116].

Though the data on sequencing for radiation–immunotherapy combination therapy is scarce, the limited evidence available in breast cancer patients demonstrates efficacy and tolerability of both concurrent and sequential regimens.

It is important to note that the increase in toxicity with the addition of immunotherapy has been attributed to the known AE profile of the chemotherapeutic agents used in many of the trials above. It will be important to follow the several ongoing studies investigating the use of combination RT-IO specific for breast cancer to further our understanding of this combination therapy’s toxicities (Table 3).

## 8. Discussion and Future Directions

Breast cancer treatment has evolved significantly from patients predominantly receiving a radical mastectomy to a more personalized approach using a variety of innovative surgical techniques, radiotherapy regimens, and systemic therapies. In this review article, we explore the ways that radiotherapy techniques have advanced in breast cancer treatment, particularly in conjunction with immunotherapy for various stages of breast cancer. Most of these trials focus on advanced or metastatic breast cancer.

Amongst the limited number of trials in early stage or locally advanced breast cancer setting, KEYNOTE-522 has been the most prominent study which has shown statistically significant higher rates of pathological complete response amongst patients that received neoadjuvant and adjuvant pembrolizumab concurrently with radiation therapy. Additional studies are currently underway to assess the utility of SBRT in a neoadjuvant setting for early-stage TNBC in hopes of increasing immunogenicity of breast cancer by promoting the release of antigens from tumor cells.

Within the setting of recurrent and metastatic breast cancer, several pilot trials have shown promising results when combining SBRT with pembrolizumab due to their suspected synergistic effects in patients with oligometastatic disease. This combination treatment is hypothesized to help with immune system sensitization. NCT03449238 is an ongoing trial assessing local control and safety of SRS treatments to brain metastases in between pembrolizumab administration. On the other hand, the non-comparative TONIC trial tests various induction strategies prior to pembrolizumab in patients with metastatic TNBC including SBRT. In this study, SBRT did not result in numerically higher response rate compared to no induction therapy, with chemotherapies doxorubicin and cisplatin resulting in the highest overall response rate. While RT did not increase overall response rate, future trials should examine synergistic effect of radiation therapy, chemotherapy, and immunotherapy with different sequencing of RT (before IO, during IO, or after IO) to determine optimal timing and effect. The importance of future trials to evaluate multi-modal treatment including combination systemic and local therapy such as RT cannot be understated. Future trials should investigate combination systemic therapy including immunotherapy and chemotherapy with and without radiation therapy and how the use of RT may affect clinical outcomes, including distant sites of disease.

Most of the studies discussed in this review focus on treating metastatic sites, rather than the primary tumor. Several key trials in other disease areas, like the DIPPER and CAPTAIN-1 trials for locally advanced and metastatic nasopharyngeal cancer, respectively, have shown that adding IO prior to treating the primary site with definitive chemoradiation improves survival in patients with a low burden of metastatic disease. Additional trials are needed in breast cancer to evaluate whether combination IO and RT for the primary site in the locoregionally advanced and metastatic setting impact survival outcomes.

Combining immunotherapy and radiation therapy has shown promise in clinical trials across multiple stages and types of breast cancer. As immunotherapy likely will continue to play a larger role in breast cancer treatment, it is crucial to optimize the treatment regimen to enhance the antitumoral response while minimizing side effects and toxicities. Current clinical trials use standard IO regimens without adjusting for potential synergy or toxicity when paired with radiation. Likewise, the ideal radiation dose and fractionation to maximize immune activation while limiting adverse effects remain undefined. Future trials should incorporate dose-finding strategies to guide safe and effective combination protocols.

While early-phase and interim analyses from these key trials have shown promising improvements across several outcomes, long-term data remains limited. Mature survival outcomes, late toxicity profiles, and durability of IO benefit have not yet been fully established in many of these studies. Continued follow-up and future analyses will be essential to clarify the sustained impact of immune checkpoint blockade in breast cancer treatment across both early-stage and metastatic settings.

As the landscape of immunotherapy and radiation therapy in breast cancer continues to evolve, there is a pressing need to identify reliable predictive biomarkers that can help guide patient selection for combined modality treatment. Tumor-infiltrating lymphocytes (TILs) and PD-L1 expression have emerged as promising candidates, particularly in TNBC where higher levels of these markers are associated with improved response to immune checkpoint inhibitors. Additionally, identifying individuals’ cancers mutation burden, may offer an avenue to identify those more likely to benefit from IO and combination IO with radiation therapy [117]. Incorporating biomarker-driven stratification into future clinical trials will be critical to identifying those patients most likely to benefit from IO–RT combinations, thereby maximizing therapeutic efficacy while minimizing unnecessary autoimmune toxicities. Furthermore, this personalized approach may help guide the optimal timing and sequencing of treatment. For patients at higher risk of irAEs, sequential rather than concurrent administration of IO and RT may be preferred. Artificial intelligence and machine learning models may help in accelerating efforts of individual biomarker analysis to develop predictive models for treatment response, timing, and toxicity [118].

As patient stratification improves, it will also be crucial to create a standardized treatment response assessment across studies. Traditional criteria such as RECIST may fail to capture unique response to IO. Future trials should adopt a standardized IO specific response criterial such as iRECIST to more accurately evaluate treatment outcomes [119].

As more innovative medications are incorporated into treatment regimens, financial toxicity will become a concern that will have to be navigated with patients and their insurance. Future combination studies should investigate the optimal timing and fractionation schedule for RT. These trials should also stratify different populations including, but not limited to age, sex, ethnicity, and gut microbiome to evaluate additional factors that can affect efficacy of immunotherapy and its effectiveness in combination with radiation.

## 9. Conclusions

Breast cancer is the most common non-cutaneous cancer diagnosed in women and is the leading cause of cancer deaths in women worldwide. Immunotherapy has emerged as a promising addition to breast cancer treatment, particularly for triple-negative and high-risk disease. While early clinical trials have demonstrated encouraging short-term outcomes when immune checkpoint inhibitors are combined with chemotherapy or radiation, several critical questions remain. Future research should prioritize the design of prospective trials that evaluate the impact of radiation therapy conjunction with immunotherapy, optimal sequencing, timing, and fractionation of radiation delivery. Dose optimization studies are particularly needed to tailor regimens that balance immune activation with toxicity. Additionally, new trials should explore the role of this combination in specific subgroups—such as patients with early-stage TNBC who may benefit from escalated therapy, and those with oligometastatic or locoregionally advanced disease where tumor control remains a key therapeutic goal. Incorporating biomarker-driven patient stratification (e.g., PD-L1 status, tumor-infiltrating lymphocytes, and BRCA mutation) and evaluating the effects of host factors such as age, microbiome, and comorbidities will be critical to optimizing outcomes. Finally, as novel systemic agents continue to evolve, it is imperative we, similarly, continue to evaluate the efficacy and safety of combined radiation therapy and immunotherapy to enhance patient-centered care, oncologic outcomes, and promote patient safety.

## Figures and Tables

**Table 2:** Summary of toxicities reported in key clinical trials for IO in breast cancer.

Trial	Key Toxicities Reported	Toxicity Notes
KEYNOTE-522	Neutropenia, fatigue, diarrhea, pneumonitis	Higher irAEs vs. control; manageable with steroids
IMpassion130	Peripheral neuropathy, neutropenia, rash	No RT; increased rash and irAEs in PD-L1+ subgroup
TONIC	Fatigue, cytokine release, hypothyroidism	Pre-treatment with SBRT increased immune response
KEYNOTE-355	Fatigue, nausea, immune-mediated AEs	No RT; highlights systemic IO safety profile

Abbreviations: AE—adverse event, RT—radiation therapy, IO—immunotherapy, SBRT—stereotactic body radiation therapy.

**Table 3:** Selected phase II or III clinical trials utilizing a combination of IO and RT for patients with breast cancer.

Trial (IO Agent [if Specified])	Phase; ClinicalTrials.gov ID	Description (Patient Population, Control Arm, Experimental Arm)	Trial Status	Expected Completion Date/Year
Radiotherapy for Extracranial Oligometastatic Breast Cancer [92]	III NCT04646564	Population: patients with extracranial oligometastatic breast cancer Control arm: Standard systemic therapy (including immunotherapy) Experimental arm: above + local radiotherapy (SBRT of 30–50 Gy in 5 fx or conventional RT of 60 Gy in 25 fx)	Recruiting	30 April 2026
SBRT Combined With PD-1 Inhibitor and Chemotherapy in Early-stage TNBC [93]	III NCT06627712	Population: patients with early-stage TNBC Control arm: PD-1 Inhibitor + chemotherapy Experimental arm: above + SBRT (24 Gy in 3 fx)	Not yet recruiting	1 November 2031
Neoadjuvant Treatment of Triple-Negative Breast Cancer with Stereotactic Radiotherapy, PD-1 Monoclonal Antibody, and Chemotherapy (pembrolizumab) [94]	II NCT06691594	Population: patients with TNBC Experimental arm (single arm study): neoadjuvant SBRT (10 Gy in 1 fx) + pembrolizumab and chemotherapy	Not yet recruiting	November 2030
Capecitabine Plus Pembrolizumab in Patients With Triple-Negative Breast Cancer After Chemo-immunotherapy and Surgery (CAPPA) (pembrolizumab) [95]	II NCT05973864	Population: patients with TNBC with residual disease after neoadjuvant chemo-immunotherapy External cohort * receiving standard of care of pembrolizumab as adjuvant therapy Experimental arm *: pembrolizumab + capecitabine as adjuvant therapy. * Local radiotherapy as per standard practice if indicated	Not yet recruiting	August 2028
Neoadjuvant Chemotherapy + PD-1 Inhibitor + Different Radiotherapy Fractionations for HR+/HER2- Breast Cancer [72]	II NCT06639672	Population: patients with HR-positive/HER2-negative breast cancer Experimental arm 1: chemotherapy + PD-1 inhibitor + RT (24 Gy in 3 fx) Experimental arm 2: chemotherapy + PD-1 inhibitor + RT (16 Gy in 1 fx) Experimental arm 3: chemotherapy + PD-1 inhibitor + RT (41.4 Gy in 15 fx) Experimental arm 4: chemotherapy + PD-1 inhibitor + RT (6–9 Gy in 12–18 fx)	Not yet recruiting	1 May 2031
Radiotherapy Followed by Chemotherapy Combined With Toripalimab in Local Advanced HR-positive, HER2-negative BC (triplimab) [96]	II NCT06705127	Population: patients with locally advanced HR-positive/HER2-negative breast cancer Experimental arm (single arm study): neoadjuvant SBRT (24 Gy in 3 fx) followed by chemotherapy + triplimab	Recruiting	1 July 2028
Investigating the Effectiveness of Stereotactic Body Radiotherapy (SBRT) in Addition to Standard of Care Treatment for Cancer That Has Spread Beyond the Original Site of Disease (PROMISE-005) [97]	II NCT03808337	Population: patients with breast cancer or NSCLC with 1–5 metastases Control arm: standard of care (may include immunotherapy) Experimental arm: above + SBRT (min dose of 30 Gy in 5 fx)	Recruiting	January 2026
SBRT, Chemotherapy, and AK104 Neoadjuvant Therapy for Triple-negative Breast Cancer (TNBC) (AK104, aka cadonilimab) [98]	II NCT06401005	Population: patients with TNBC Experimental arm (single arm): SBRT (24 Gy in 3 fx or 18 Gy in 3 fx), then chemotherapy + cadonilimab, then surgery, then adjuvant immunotherapy with or without adjuvant radiotherapy	Recruiting	1 September 2027
SBRT, Chemotherapy, and AK112 Neoadjuvant Therapy for Luminal-type Breast Cancer (AK112, aka Ivonescimab) [99]	II NCT06402435	Population: patients with luminal-type breast cancer Experimental arm (single arm): SBRT (24 Gy in 3 fx or 18 Gy in 3 fx), then chemotherapy + cadonilimab, then surgery	Recruiting	1 September 2027
A Study of Radiation Therapy With Pembrolizumab and Olaparib or Other Radiosensitizers in Women Who Have Triple-Negative or Hormone Receptor-Positive/Her2 Negative Breast Cancer (pembrolizumab) [85]	II NCT04683679	Population: patients with metastatic TNBC or HR-positive/HER2-negative breast cancer Experimental arm A: pembrolizumab + olaparib + RT (24–27 Gy in 3 fx or 30 Gy in 5 fx for large tumors) Experimental arm B: above without olaparib	Recruiting	January 2026

Abbreviations: fx—fraction, Gy—Gray, NSCLC—non-small cell lung cancer, PD-1—programmed cell death protein 1.

## Data Availability

No new data were created or analyzed in this study. Data sharing is not applicable to this article.

## Abbreviations

AE	Adverse Event
APC	Antigen-Presenting Cell
CA	Cancer
CI	Confidence Interval
CPS	Combined Positive Score
DFS	Disease-Free Survival
EBCTCG	Early Breast Cancer Trialists’ Collaborative Group
EFS	Event-Free Survival
FAST	Fractionation of Adjuvant Radiotherapy in Breast Cancer
fx	Fraction
Gy	Gray
HER2	Human Epidermal Growth Factor Receptor 2
HF	Hypofractionated
HR	Hormone Receptor/Hazard Ratio
ICD	Immunogenic Cell Death
ICI	Immune Checkpoint Inhibitor
IMPORT	Intensity-Modulated Partial Breast Radiotherapy
IO	Immunotherapy
IORT	Intraoperative Radiation Therapy
irAEs	Immune-Related Adverse Events
MHC	Major Histocompatibility Complex
NCT	National Clinical Trial
NSABP	National Surgical Adjuvant Breast and Bowel Project
OS	Overall Survival
PBI	Partial Breast Irradiation
pCR	Pathologic Complete Response
PD-1	Programmed Death-1
PDL-1	Programmed Death Ligand 1
PFS	Progression-Free Survival
RAPID	Randomized Trial of Accelerated Partial Breast Irradiation
RT	Radiation Therapy
SBRT	Stereotactic Body Radiation Therapy
SRS	Stereotactic Radiosurgery
TILs	Tumor-infiltrating lymphocytes
TNBC	Triple-Negative Breast Cancer
WBI	Whole Breast Irradiation
